# Incidence and immunomic features of apyretic COVID-19 in patients affected by solid tumors: a prospective cohort study

**DOI:** 10.1186/s12967-022-03429-0

**Published:** 2022-05-14

**Authors:** Francesco Ravera, Roberto Borea, Gabriella Cirmena, Martina Dameri, Lorenzo Ferrando, Maurizio Gallo, Cecilia Casini, Neri Fallani, Mario Stabile, Valentina Barbero, Roberto Murialdo, Lucia Tixi, Margherita Cappuccio, Andrea Cuboni, Irene Sivieri, Giuseppe Fornarini, Andrea De Maria, Alberto Ballestrero, Gabriele Zoppoli

**Affiliations:** 1 Department of Internal Medicine (DiMI), Università Degli Studi Di Genova, Genoa, Italy; 2 IRCCS Ospedale Policlinico San Martino, Genoa, Italy; 3 Department of Health Sciences (DISSAL), Università Degli Studi Di Genova, Genoa, Italy

**Keywords:** COVID-19, Asymptomatic infection, Serology, Cancer, Transcriptome sequencing

## Abstract

**Background and rationale:**

Little is known about SARS-CoV-2 seroconversion in asymptomatic patients affected by solid cancer, and whether it is associated with specific transcriptomics changes in peripheral blood mononuclear cells (PBMC).

**Methods:**

Patients affected by solid cancer treated in a top comprehensive cancer center in Italy during the first COVID-19 pandemic wave, and negative for COVID-19-symptoms since the first detection of COVID-19 in Italy, were prospectively evaluated by SARS-CoV-2 serology in the period between April 14th and June 23rd 2020. Follow-up serologies were performed, every 21–28 days, until August 23rd 2020. All SARS-CoV-2 IgM + patients underwent confirmatory nasopharyngeal swab (NPS). PBMCs from a subset of SARS-CoV-2 IgM + patients were collected at baseline, at 2 months, and at 7 months for transcriptome sequencing.

**Results:**

SARS-CoV-2 serology was performed on 446 of the 466 recruited patients. A total of 14 patients (3.14%) tested positive for at least one SARS-CoV-2 immunoglobulin in the period between April 14th and August 23rd 2020. Incidence of SARS-CoV-2 IgM decreased from 1.48% in the first month of the accrual to 0% in the last month. Viral RNA could not be detected in any of the NPS. PBMC serial transcriptomic analysis showed progressive downregulation of interleukin 6 upregulated signatures, chemokine-mediated signaling and chemokine-chemokine receptor KEGG pathways. B- and T-cell receptor pathways (p-values = 0.0002 and 0.017 respectively) were progressively upregulated.

**Conclusions:**

SARS-CoV-2 seroconversion rate in asymptomatic patients affected by solid cancer is consistent with that of asymptomatic COVID-19 assessed in the general population through NPS at the peak of the first wave. Transcriptomic features over time in IgM + asymptomatic cases are suggestive of previous viral exposure.

**Supplementary Information:**

The online version contains supplementary material available at 10.1186/s12967-022-03429-0.

## Introduction

The global emergency caused by Coronavirus Disease 2019 (COVID-19) brought substantial changes in the approach to infective and non-infective diseases. Massive testing coupled with contact tracing performed on individuals presenting a high risk of contracting the infection or suffering of serious complications from it, such as health care workers or nursing homes residents respectively, is one of the main strategies carried out to limit the spread of the severe acute respiratory syndrome-coronavirus 2 (SARS-CoV-2). Asymptomatic infections represent one of the main challenges for the pursuit of this intent, given the difficulties typically encountered in performing extensive and effective screening tests in order to identify and isolate asymptomatic carriers, preventing the spread of SARS-CoV-2 to susceptible individuals.

The study of the infection dynamics and the immune reaction in asymptomatic COVID-19 patients is of great interest. Asymptomatic carriers identified through real-time reverse-transcriptase polymerase chain reaction (RT-PCR) assay performed on nasopharyngeal swab (NPS) seem to yield a viral load comparable to symptomatic patients [1], with a relevant potential of transmission which must be considered in order to perform effective public health strategies [2]. Moreover, even though most asymptomatic COVID-19 patients develop neutralizing antibodies, an average lower rate of high antibody titer production is reported in comparison to severe infections [3]. Several works suggest that NPS alone presents low sensitivity in detecting asymptomatic infections, being outperformed by the association of NPS and serology [4]. COVID-19 has had a major impact on the management of chronically ill patients, with patients affected by neoplastic disorders being amongst the most negatively impacted categories by the pandemic. In particular, delays or cancellation of surgery and early interruption of palliative treatment are widely reported, with substantial consequences on patients’ prognosis and quality of life [5].

The study of the incidence of asymptomatic infection along with the immune response and clinical features of patients affected by solid tumors is particularly relevant in a pandemic setting, given the necessity for these patients to prosecute treatments typically administered in dedicated facilities.

The aim of the present study is to estimate the incidence of asymptomatic infections, together with the prevalence of SARS-CoV-2 immunoglobulins, in patients affected by solid tumors who had access to the Cancer Center of a large tertiary care hospital during the first wave of COVID-19 in North-West Italy. Infection incidence was investigated by serological testing. Infected patients were identified as SARS-CoV-2 IgM and/or IgM and IgG positive and underwent confirmatory NPS. Serology was repeated with a periodicity of at least 21 days for each patient, in order to detect new seroconversions occurring after the first test and monitor variations in the immunologic setting of detected positive patients.

Moreover, in order to study transcriptomic changes associated with the seroconversion of SARS-CoV-2 immunoglobulins, peripheral blood mononuclear cells (PBMC) signatures were evaluated through sequential RNA sequencing on RNA extracted from blood samples collected at diagnosis and in the follow-up period from two patients testing positive for SARS-CoV-2 serology.

## Patients and methods

### Ethics approval and consent to participate

This study was approved by the local Ethics Committee (Comitato Etico Regione Liguria, reference number: 10575). Written informed consents were obtained from all study participants, and all methods were carried out in accordance with relevant guidelines and regulations.

### Study population

Patients included in this study were selected upon the following inclusion and exclusion criteria. Inclusion criteria were: informed written consent; diagnosis of solid tumor in any stage, undergoing active treatment; outpatient access for an active, scheduled treatment to U01H and H04 units of the Cancer Center of Ospedale Policlinico San Martino; corporeal temperature < 37 °C when accessing the Cancer Center facility.

Exclusion criteria were: diagnosis of hematologic malignancy in the five years preceding the study; previously confirmed SARS-CoV-2 infection; CT elevation > 37 °C when accessing the Cancer Center facility; COVID-19 suggestive symptoms in the period following the first confirmed case in Italy, i.e. February 21st 2020. Suggestive COVID-19 symptoms included fatigue, dry cough, anorexia, myalgias, dyspnea, sputum production, fever (> 38 °C) and low-grade fever (37–38 °C), anosmia, dysgeusia, headache, sore throat, rhinorrhea, suggestive gastrointestinal symptoms, suggestive dermatologic findings.

As per hospital protocol, outpatients were allowed access to the Cancer Center facilities only after CT measurement < 37 °C and the fulfillment of a questionnaire which investigated possible contacts with infected individuals and suggestive COVID-19 symptoms over the previous two weeks. Surgical or FFP2 masks were mandatory for both patients and health care workers in the Cancer Center during the entire period of the study. Type and stage of cancer, along with ongoing therapy and comorbidities were recorded for all enrolled patients.

### Study design: patients’ accrual and follow-up

**Fig. 1:**
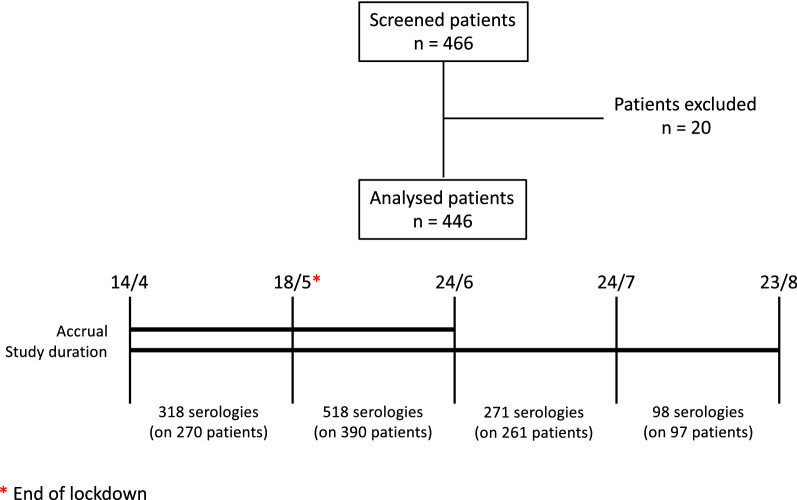
Study diagram. Patients were recruited in the period between April 14th and June 23rd. May 18th corresponds to the end of lockdown measures in Italy. Follow-up serologies were performed until August 23rd at a distance of at least 21 days from each other

Patients were recruited in the period from April 14th to June 23rd 2020. Extensive serology was performed until August 23rd 2020. Follow-up serologies were performed for each patient at a distance of at least 21 days from each other (Fig. 1). Confirmatory NPS was performed at the first positivity for SARS-CoV-2 IgM. In case of SARS-CoV-2 IgM and/or IgG detection after the first serology, patients were investigated for COVID-19 suggestive symptoms over the previous months. Complete blood count (CBC) was assessed concomitantly with the first detection of SARS-CoV-2 IgM or IgG.

### SARS-CoV-2 serology

SARS-CoV-2 IgM and IgG were assessed through chemiluminescence immunoassay (CLIA) kits, supplied by Shenzhen YHLO Biotech Co., Ltd (China). Magnetic beads of these CLIA assays are coated with two antigens of SARS-CoV-2 (nucleocapsid protein or N protein, spike protein or S protein). All serum antibody tests were performed with an iFlash 1800 fully automated CLIA analyzer from Shenzhen YHLO Biotech Co., Ltd (China).

### SARS-CoV-2 RNA assessment on NPS

Confirmed infection was defined as RT-PCR positive from a nasal and/or throat swab or BALF according to World Health Organization interim guidance [6].

SARS-CoV-2 RNA detection on NPS was performed using RT-PCR targeted at open reading frame 1ab and nucleocapsid protein genes. RNA was extracted from the samples and then underwent RT-PCR with SARS-CoV-2-specific primers and probes. A cycle threshold (Ct) value of less than 37 was defined as a positive test, and a Ct value of 40 or more was considered as a negative test. An equivocal result, defined as a Ct value between 37 and 40, required confirmation by retesting. If the repeated Ct value was less than 40 and an obvious peak was observed, or if the repeated Ct value was less than 37, the result was deemed positive.

### Exploratory aim: transcriptome sequencing

Blood samples (3 mL each) were collected at 3 different time points from two patients who tested positive for SARS-CoV-2 IgM, remaining asymptomatic for all the course of seven months and repeatedly negative for molecular testing, in order to perform PBMC transcriptome sequencing. Blood was collected in Applied Biosystems Tempus Tubes, prefilled with 6 ml of preserving and virus-inactivating buffer, and stored at − 80 °C. RNA was isolated by using Tempus™ Spin RNA Isolation Kit (Thermo Fisher) following the manufacturer’s recommended protocol.

We determined RNA yield and purity based on 260/280 and 260/230 absorbance ratios with a NanoDrop ND-1000 spectrophotometer (Thermo Fisher). RNA integrity number was assessed using a Tapestation 2200 System (Agilent Technologies). Concerning the library preparation, RNA was reverse-transcribed by using the SuperScript^®^ VILO™ cDNA Synthesis kit (Thermo Fisher) on 50 ng of total RNA. Complementary DNA was then amplified by using Ion AmpliSeq™ Transcriptome Human Gene Expression Chef-Ready Kit (Cat. No. A31446), which allows simultaneous gene expression measurement of over 20,000 human Refseq genes. This panel contains 41,604 primers, approximately 150 bases in length, in a single pool. The barcoded libraries were prepared on the Ion Chef ™ system (Cat. No. A27759) (ThermoFisher Scientific). Library size was checked using High Sensitivity DNA ScreenTape (Agilent) on a Tapestation 2200 device, while library concentration was evaluated with a Qubit^®^ 2.0 Fluorometer using Qubit™ dsDNA HS Assay Kit. Each barcoded library was diluted into 50 pM concentrations for template preparation and sequenced on an Ion 540™ chip (Cat. No. A27766) on the Ion S5 System, according to the manufacturer's protocol [7].

Gene expression analysis was performed in the R environment. Raw counts were imported using the edgeR package. Scaling of raw library size was used to calculate normalization factors. The normalization method used was upper quantile [8], in which the scale factors are calculated from 75% quantile of the counts for each library. The Gene set enrichment analysis was carried out using the GSVA R package, with the gsva method and Poisson distribution. The C7 (immunologic signatures) gene set was obtained from MSigDB (v.7) [9].

Only pathways displaying an increasing/decreasing monotonic profile across all time points were selected for the transcriptional analysis. Finally, we selected the top 5 enriched/depleted immune pathways with the highest variance across samples. KEGG and Gene Ontology pathway enrichment analyses were performed using DAVID v6.8 [10, 11]. Uninominal p-values and not false discovery rates were reported, due to the very low number of transcriptome samples and the exploratory nature of the analysis.

Additionally, we performed CIBERSORT in absolute mode using transcripts per million levels allowing to deconvolute the immune subpopulations (LM22) on samples collected at the three time points from the two selected patients [12]. We used ComplexHeatmap to perform clustering with default settings [13].

## Results

### Demographics

A total of 466 patients accessing U01H and H04 units of Ospedale Policlinico San Martino Cancer Center were screened in the period between April 14th and June 23rd 2020 with the inclusion and exclusion criteria outlined above before undergoing the first SARS-CoV-2 serology, which was performed on 446 individuals. Of these, 146 were affected by breast cancer, 103 by colorectal cancer, 42 by prostate cancer, 28 by pancreatic cancer, 25 by ovarian cancer, 18 by renal cancer, 17 by GIST, 14 by gastric cancer, 12 by hepatobiliary cancer, 11 by lung cancer, 8 by sarcoma, 5 by endometrial cancer, 4 by cervical cancer, 3 by esophageal cancer, 3 by urothelial cancer, and 7 by other neoplasms. All patients were diagnosed with cancer before January 2020.

Twenty patients were ruled out (6 because of COVID-19 suggestive symptoms occurred in the period preceding the date of enrollment, 6 because of hematologic malignancy, 1 because of previous NPS-confirmed SARS-CoV-2 detection, 7 because of antineoplastic therapy interruption or prosecution in domiciliary regimen).

Patients’ age ranged between 27 and 90 (9 patients under 40, 30 patients between 40 and 50, 92 patients between 50 and 60, 139 between 60 and 70, 135 between 70 and 80, and 40 between 80 and 90).

Of the recruited patients, 397 underwent at least two serologies, 312 underwent at least three serologies, and 122 underwent four serologies.

### The end of lock-down measures did not cause an increase of SARS-CoV-2 seroincidence in oncologic outpatients

A total of 14 patients (3.14%) tested positive for at least one SARS-CoV-2 immunoglobulin in the period between April 14th and 23th August 2020. See Table 1 for serology results in the accrual and follow-up phases.

**Table 1:** Serological profile of patients detected as positive for SARS-CoV-2 immunoglobulins in the period between April 14th and August 23rd

UPN	Date First Serology	IgM	IgG	Date Second Serology	IgM	IgG	Date Third Serology	IgM	IgG	Date Fourth Serology	IgM	IgG	Date Fifth Serology	IgM	IgG	Date Swab	Result Swab
1	22–04–2020	+	–	20–05–2020	+	–	17–06–2020	+	–	16–07–2020	+	–	17–12–2020	+	–	13–05–2020	–
2	23–04–2020	W + ^a^	–	18–06–2020	+	–										30–04–2020	–
3	29–04–2020	–	–	22–05–2020	W + ^a^	–	19–06–2020	–	–							27–05–2020	–
4	02–05–2020	–	+	27–05–2020		+	26–06–2020	–	+								
5	13–05–2020	+	+	03–06–2020	+	+	25–06–2020	ND^a^	+							20–05–2020	–
6	14–05–2020	–	+	08–06–2020	–	+											
7	14–05–2020	–	W + ^a^														
8	15–05–2020	+	+	05–06–2020	+	+	17–07–2020	+	+	16–07–2020	+	+	11–12–2020	–	+	21–05–2020	–
9	22–05–2020	–	W + ^a^	19–06–2020	–	+	17–07–2020	–	ND^a^								
10	22–05–2020	+	–	17–07–2020	+	–	14–08–2020	+	–							25–05–2020	–
11	25–05–2020	–	+	22–06–2020	–	+	20–07–2020	–	+								
12	27–05–2020	+	–	26–06–2020	+	–	27–07–2020	+	–							28–05–2020	–
13	01–06–2020	–	–	29–06–2020	–	+	27–07–2020	–	+								
14	04–06–2020	–	+														

Both accrual and follow-up serologies are reported. W + stands for weakly positive result, while ND for undetermined result

In the first month of the accrual, from April 14th to May 17th, SARS-CoV-2 serology was performed on 270 patients, of which 48 underwent a second serology in the same period. SARS-CoV-2 IgM were detected in 4 patients (1.48% of patients, 1.26% of serologies), of which 2 positive for IgM only and 2 for both IgM and IgG. Three patients tested positive for SARS-CoV-2 IgG only (1 weakly positive).

In the second month of the accrual, from May 18th to June 23rd, SARS-CoV-2 serology was performed on 390 patients, of which 134 underwent a second serology in the same period. SARS-CoV-2 IgM were detected in 2 patients (0.51% of patients, 0.38% of serologies), both negative for SARS-CoV-2 IgG. SARS-CoV-2 IgG were detected in 3 patients. One patient (UPN3), who tested weakly positive for SARS-CoV-2 IgM and negative at the following serology, was deemed as a false positive result and was therefore excluded from the count.

In the third month of the study, from June 24th to July 23rd, SARS-CoV-2 serology was performed on 261 patients, of which 18 underwent a second serology in the same period. No patients tested positive for SARS-CoV-2 IgM, while IgG were detected in 1 patient.

In the fourth month of the study, from July 24th to August 23rd, SARS-CoV-2 serology was performed on 97 patients, of which 1 underwent a second serology in the same period. No SARS-CoV-2 immunoglobulins were detected in this period.

NPS was performed after serology on all 7 patients detected as positive for SARS-CoV-2 IgM (see Table 1). None of the NPS detected viral RNA.

Complete data concerning clinical features and CBC of patients detected as positive for SARS-CoV-2 immunoglobulins are reported in Additional file 4: Table S1. In general, we observed that IgM + patients presented higher Neutrophils count compared to IgG-only + patients (median Neutrophils count in IgM + patients = 3295/µL, 2.5 − 97.5 quantile interval = 1210/µL − 4675/µL; median Neutrophils count in IgM- patients = 2000/µL, 2.5 − 97.5 quantile interval = 1124.5/µL − 4014/µL, p = 0.39). However, the limited sample size and the presence of multiple confounding factors do not allow to draw significant conclusions from these findings.

Overall, these results point out that the end of lockdown measures, which occurred in Italy on May 18th 2020, did not cause a new increase of COVID-19 incidence in patients affected by solid tumors, in accord with data of COVID-19 incidence in the general population [14].

### COVID-19 elicits a multiform serological response in patients affected by solid tumors

Of the 11 patients detected as positive for SARS-CoV-2 immunoglobulins at the first serology, two underwent three follow-up serologies, six underwent two follow-up serologies, and two underwent one follow-up serology (Table 1). Two patients did not undergo follow-up serologies.

UPN8 maintained the positivity for both antibodies across all tests, while UPN5 lost the IgM positivity at the fourth serology. Four IgM + patients (UPN1, UPN2, UPN10, UPN12) did not undergo IgG seroconversion. Two patients (UPN4, UPN11) detected as IgG+ at the first serology maintained the IgG positivity at the second and third test. Among those patients who resulted negative at the first serology, UPN3 tested weakly positive for SARS-CoV-2 IgM only at the second serology, indicating a false positive result, while UPN13 tested positive for SARS-CoV-2 IgG at the second and third serology.

### PBMCs transcriptomic features assessed in asymptomatic patients affected by solid tumor with serological evidence of SARS-Cov2 are consistent with a previous viral infection

Blood samples (3 ml each) were collected from two SARS-CoV-2 IgM positive patients (UPN 1 and UPN 8) affected by advanced breast cancer (stage IV and IIIB, respectively) at three different time points (Baseline, at 2 months, at 7 months). Both patients underwent a further serology at the moment of the last blood collection. One patient (UPN 1) showed IgM seroconversion at baseline, and never developed IgG, albeit maintaining IgM positivity at all time points, whereas the other (UPN 8) showed seroconversion to IgG at all time points with disappearance of IgM at the last blood draw. Neither patient tested positive for SARS-CoV-2 NPS at any time point. Both were consistently asymptomatic throughout the collection period.

The top 5 enriched and depleted immune signatures with the highest variance across samples were selected from 630 and 44 monotonic signatures respectively.

**Fig. 2:**
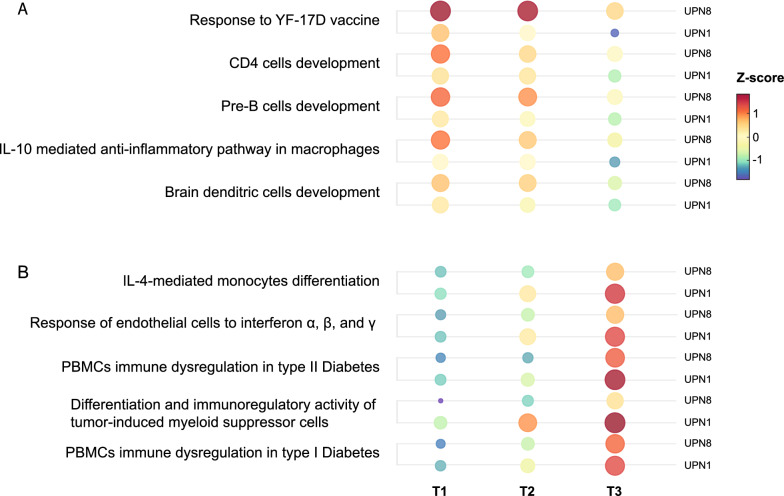
PBMCs transcriptomic profile assessed on peripheral blood samples collected from two positive patients for SARS-CoV-2 IgM at baseline (T1), 2 months (T2), and 7 months (T3) from the first serology. The top 5 depleted (**A**) and enriched (**B**) immunologic signatures with the highest variance across samples were selected for the transcriptomic analysis

The top 5 downregulated signatures (Fig. 2A) included genes pertaining to the immune response to Yellow Fever vaccine YF-17D (GSE13485), IL-10 mediated anti-inflammatory pathway in macrophages (GSE9509), and the development of pre-B cells (GSE24814), brain dendritic cells (GSE29949), and CD4 cells (GSE8835).

The top 5 upregulated signatures (Fig. 2B) included genes pertaining to the IL4-mediated monocytes differentiation (GSE16385 signature), myelo-monocytic differentiation and immunoregulatory activity of tumor-induced myeloid suppressor cells (GSE21927 signature), response of endothelial cells to interferon α, β, and γ (GSE3920 signature), and PBMCs immune dysregulation in type I and II diabetes (GSE9006).

**Fig. 3:**
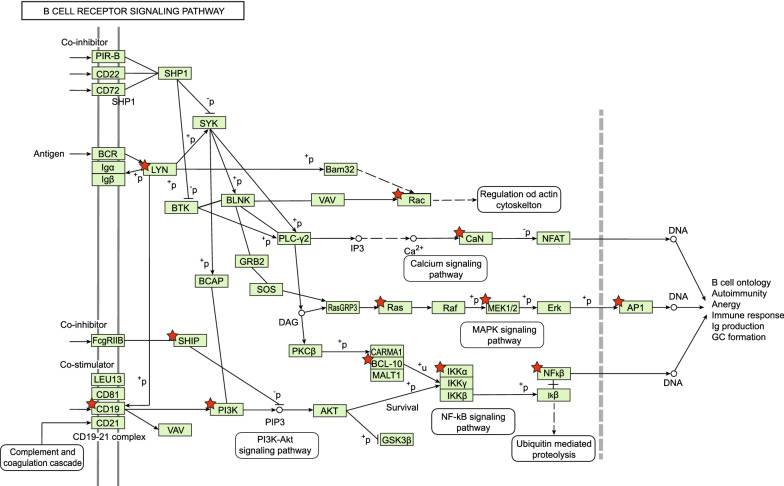
KEGG enrichment analysis of genes pertaining to the B-cell receptor signaling pathway in the RNA extracted from PBMCs collected from two SARS-CoV-2 IgM+ patients. Genes encoding for the star-marked elements, including LYN, SHIP, CD81, PI3K, Rac, CaN, Ras, BCL-10, MEK 1/2, IKKα, IP1, and NFkB, and implied in the B-cell receptor signaling pathway, were found to be increasingly upregulated in these patients over time (p-value = 0.0002)

KEGG enrichment analysis of the top downregulated signatures identified genes pertaining to chemokine-mediated signaling pathways, such as CCL20, CXCL12, and CXCR1, which were found overexpressed at the first blood draw and gradually decreased over time, as did several genes of the chemokine-chemokine receptor KEGG pathway (p-value = 0.018, see Additional file 1: Fig. S1), such as CXCR6, IL4, LIF, OSM, IL6ST, IL12RB2 – part of the Interleukin 6/12-like signaling. On the other hand, KEGG enrichment analysis of the top upregulated signatures identified genes involved in the B-cell receptor pathway (p-value = 0.0002, see Fig. 3) and T-cell receptor pathway (p-value = 0.017, see Additional file 2: Fig. S2).

The CIBERSORT analysis showed a progressive decrease of neutrophils enrichment score and an increase of CD8 cells enrichment in both patients (see Additional file 3: Fig. S3). The other immune cells populations did not show significant variations across the investigated time points.

Overall, the observed behavior is consistent with an early upregulation of immune-related transcripts – likely induced by a viral infection – followed by their normalization over the course of a few months. At the same time, such findings suggest the progressive development of B- and T-cell mediated immune response towards the likely initial infectious stimulus.

## Discussion

Neoplastic patients represent a peculiar population in the panorama of COVID-19 pandemic, as they are exposed to both the immunosuppressive and immunostimulating effects of the neoplasm itself and the systemic treatment, with a higher risk of developing a severe SARS-CoV-2 infection compared to healthy individuals [15]. At the same time, however, immunosuppressive agents and ACE-inhibitors, often administered concurrently with the antineoplastic therapy, are proved to interact with the so-called cytokine and bradykinin storms, which are imputed to be at least partially responsible for severe infections, altering the clinical response to the virus and resulting in milder or absent symptoms compared to non-treated patients in particular sub-settings [15]. All these elements make the detection of asymptomatic carriers of SARS-CoV-2 in this population particularly challenging.

The Cancer Center of Ospedale Policlinico San Martino is one of the major Italian facilities for cancer treatment. Over the initial few weeks of COVID-19 outbreak, periodical serologic testing for SARS-CoV-2 specific class IgM and IgG was performed by practicing oncologists, although without a fixed or agreed upon schedule. A preliminary series of 21 cases referring to the Cancer Center, obtained between April 14th and April 24th, was able to identify one apyretic case of a patient affected by a solid tumor with SARS-CoV-2 specific positive serology (both IgM and IgG).

In order to have a realistic estimate of the incidence rate of asymptomatic infections in oncologic patients during the first wave of COVID-19, serological tests were systematically performed on asymptomatic neoplastic patients who had access to two of the clinical units of the Cancer Center, screening for the occurrence of COVID-19 suggestive symptoms in the period following the first diagnosis of COVID-19 in Italy (i.e. February 21st 2020). At the time of this study an extensive NPS-based testing was hardly applicable for screening procedures on a large population, this being mainly reserved to confirm suspect cases of infection or to monitor health care workers exposed to relevant risks of infection on daily bases. NPS was therefore reserved to those patients who resulted positive for SARS-CoV-2 IgM, the latter undergoing seroconversion before IgG and declining after about a month from the infection [16], being typically reported as more suitable for the detection of acute or recent infections.

None of the patients ever tested positive for COVID-19 before seroconversion or either reported suggestive symptoms in the period following its first detection in Italy. Of note, none of the NPS performed on SARS-CoV-2 IgM + patients was able to detect viral RNA. Reported seroconversions are therefore consequence of asymptomatic infections and the absence of detectable viral RNA is indeed consistent with the substantial reduction of SARS-CoV-2 viral load typically occurring after the development of B and T cells mediated immunity [17]. The persistence of IgM positivity across the follow-up serologies without IgG seroconversion, observed in four patients, may reflect a peculiar immune response to epithelium-dwelling nidovirales, that entices an isolated IgM response with absent isotypic switch, in line with data shown by Zhao et al. [18]. These observations may also imply an impaired immune reaction to the virus in asymptomatic neoplastic patients undergoing systemic treatment, and are apparently in contrast with data shown by Thakkar et al., who report a 92% IgG seroconversion rate after infection [19]. The latter study, however, mostly involved patients affected by cancer diagnosed with symptomatic COVID-19, all with previous PCR-confirmed detection of SARS-CoV-2. The observed seroconversions may also be explained to some extent as the result of cross-reactivity with other human coronaviruses. The assay used does not distinguish in fact between N- or S- protein reactivity, and early detection assays were generally burdened by relevant cross-reactivity rates, especially for N protein.

According to our findings, an isolated IgM positivity cannot be considered as an accurate indicator of acute or recent infection. Nevertheless, the rate of SARS-CoV-2 IgM detection (1.48% of patients) in the first month of accrual in asymptomatic neoplastic patients is consistent with the rate of asymptomatic infections diagnosed after massive testing via NPS in particular settings such as Vo’ Euganeo during the peak of the first wave [14]. The end of lockdown measures occurred in Italy on May 18th did not cause an increase of asymptomatic COVID-19, as proved by the progressive decrease of SARS-CoV-2 IgM detection rate (0.51% over the second month, 0% over the third and fourth month of the study). None of the patients detected as positive for SARS-CoV-2 immunoglobulins was further diagnosed with symptomatic or asymptomatic COVID-19 before SARS-CoV-2 vaccines were available.

Despite the inability of detecting SARS-CoV-2 RNA on any NPS, the serial analysis of PBMC transcriptomic signatures conducted on two asymptomatic patients positive for SARS-CoV-2 IgM revealed, in both IgG positive and negative patients, a trend consistent with an initial immune perturbation that occurred before the first serology. The analysis of the top depleted immunologic signatures, with particular concern to the signature associated with the immune response to YF-17D vaccine, showed indeed a downregulation of genes typically upregulated by IL6, reported as overexpressed in COVID-19 with implications concerning the severity of the disease [20]. Also, a progressive decrease in the expression of genes belonging to chemokine-mediated signaling pathways, such as CCL20, CXCL12, and CXCR1, together with genes of the chemokine-chemokine receptor KEGG pathway was observed. On the other hand, the analysis of the top enriched immunologic signatures showed an upregulation of genes implied in both B-cell receptor and T-cell receptor pathways. Taken together, these observations are suggestive of an immunostimulating event that occurred before the first test, followed by a subsequent normalization in the expression of genes related to a likely infection-mediated inflammation, in parallel with the development of B cells and T cells mediated immunity toward the infective agent. Moreover, this is consistent with the progressive decrease of neutrophils enrichment score and the increase of CD8+ cells score showed by the CIBERSORT analysis, which corroborates the occurrence of a previous acute inflammatory event.

Such findings are generally concordant with data shown by Yu et al. who illustrate distinctive immune signatures able to discriminate SARS-CoV-2 + individuals with different clinical manifestations [21]. In particular, a significantly different expression of transcripts pertaining to macrophage activation, response to interferon, and chemokine pathways is described between asymptomatic and pre-symptomatic SARS-CoV-2 + patients, consistently with those identified in the present work. Our exploratory analysis, however, is not easily comparable with other works based on case–control experiments, in part limiting the relevance of such a comparison.

The present study is burdened by several limitations. The detection of SARS-CoV-2 IgM in patients anamnestically screened as asymptomatic since the first detection of a COVID-19 case in Italy does not allow to exclude the occurrence of a symptomatic infection before that period, especially if no NPS detected viral RNA at the time of the first positive serology. Confirmatory NPS were performed in some cases several days after the detection of SARS-CoV-2 IgM, reducing the possibility of detecting viral RNA even in those patients presenting a detectable viral load after seroconversion. Serological tests performed through CLIA present suboptimal sensitivity (around 70%) but high specificity (above 90%) [22]. The IgM weak positivity in UPN3 is reasonably the only false positive result. Finally, the approach outlined above is hardly replicable in a global setting where the rate of vaccinated people is increasing.

Concerning the transcriptomic analysis of PBMCs, the outlined findings, despite suggestive, are obtained from a restricted cohort of patients affected by breast cancer and undergoing systemic treatment. Such a condition potentially represents indeed a relevant confounding factor, especially in regard to the transcriptomic signature of tumor-induced myeloid suppressor cells (GSE21927) and the overall upregulation of genes involved in B- and T-cell receptors pathways. Myeloid suppressor cells seem in fact to be positively correlated with tumor burden, and the administration of systemic treatment may further augment their concentration and activity in those patients who achieve a suboptimal response to chemotherapy [23]. On the other hand, B cell activity in patients affected by breast cancer undergoing chemotherapy is typically enhanced and can lead to diverse effects, eliciting either anti-tumor T cell immunity or, conversely, the switch of CD4+ T cells to T-regulatory cells with immunosuppressive effects [24, 25]. However, the two cases were treated with very different drugs, namely Epirubicin and a CDK4/6 inhibitor. Since we report only on the shared monotonic alterations of both patients, it seems unlikely that these are associated with treatment. Since we are not aware of publicly available datasets containing gene expression data of serially collected PBMC from patients affected by breast cancer undergoing cyclin-dependent kinase inhibitors or anthracyclines for a robust comparison with our results, we cannot draw direct comparison with the available signatures. A proper assessment of confounding factors would require larger cohorts of patients, which is outside the purpose of this exploratory analysis.

## Conclusions

Even though the effective rate of asymptomatic COVID-19 in patients affected by solid tumors is suboptimally assessed through SARS-CoV-2 serology, as no NPS actually detected viral RNA in any IgM+ patient, the seroprevalence of SARS-CoV-2 IgM in this population reflects the incidence of asymptomatic COVID-19 in the general population during the first wave of COVID-19 pandemic. The assessment of PBMC transcriptomic profile supports the effective occurrence of a viral infection, even in case of complete absence of symptoms, and may be exploited in future for clinical purposes, such as the development of predictive transcriptional signatures from peripheral blood of de novo diagnosed patients [26].

## Supplementary Information

**Additional file 1: Figure S1.** KEGG enrichment analysis of genes pertaining to the chemokine-mediated signaling pathways in RNA extracted from PBMCs collected from two positive patients for SARS-Cov-2 IgM. Genes coding for the star-marked elements, implied in various chemokine-mediated and chemokine-chemokine signaling pathways, were found overexpressed at the first blood draw and gradually decreased over time (p-value = 0.018).

**Additional file 2: Figure S2. **KEGG enrichment analysis of genes pertaining to the T-cell receptor signaling pathway in RNA extracted from PBMCs collected from two positive patients for SARS-Cov-2 IgM. Genes coding for the star-marked elements, implied in the T-cell receptor signaling pathway, were found to be increasingly upregulated in these patients over time (p-value = 0.017).

**Additional file 3: Figure S3.** CYBERSORT analysis of PBMCs transcripts of samples collected from the two selected patients at baseline (T1), 2 months (T2), and 7 months (T3).

**Additional file 4: Table S1.** Clinical data and CBC of patients detected as positive for SARS-CoV-2 immunoglobulins. Age, sex, type and stage of cancer, along with type, dosage, and cycles of chemotherapy are reported. Pre-chemotherapy steroids were repeatedly administered at each cycle of chemotherapy in the form of 8–10 mg of dexamethasone.

## Data Availability

The datasets used and analyzed during the current study are available from the corresponding author on reasonable request.

## Abbreviations

COVID-19: Coronavirus Disease 2019
SARS-CoV-2: Severe acute respiratory syndrome-coronavirus 2
RT-PCR: Reverse-transcriptase polymerase chain reaction
NPS: Nasopharyngeal swab
PBMC: Peripheral blood mononuclear cells
CBC: Complete blood count
CLIA: Chemiluminescence immunoassay
Ct: Cycle threshold
FGF23: Fibroblast growth factor 23
PTGIS: Prostaglandin I2 synthase
IL6: Interleukin-6
