# Supplementary figures and images for: Incidence and immunomic features of apyretic COVID-19 in patients affected by solid tumors: a prospective cohort study
Source: J Transl Med. 2022 May 14;20:230. doi: 10.1186/s12967-022-03429-0 (PMC9107211; doi:10.1186/s12967-022-03429-0)

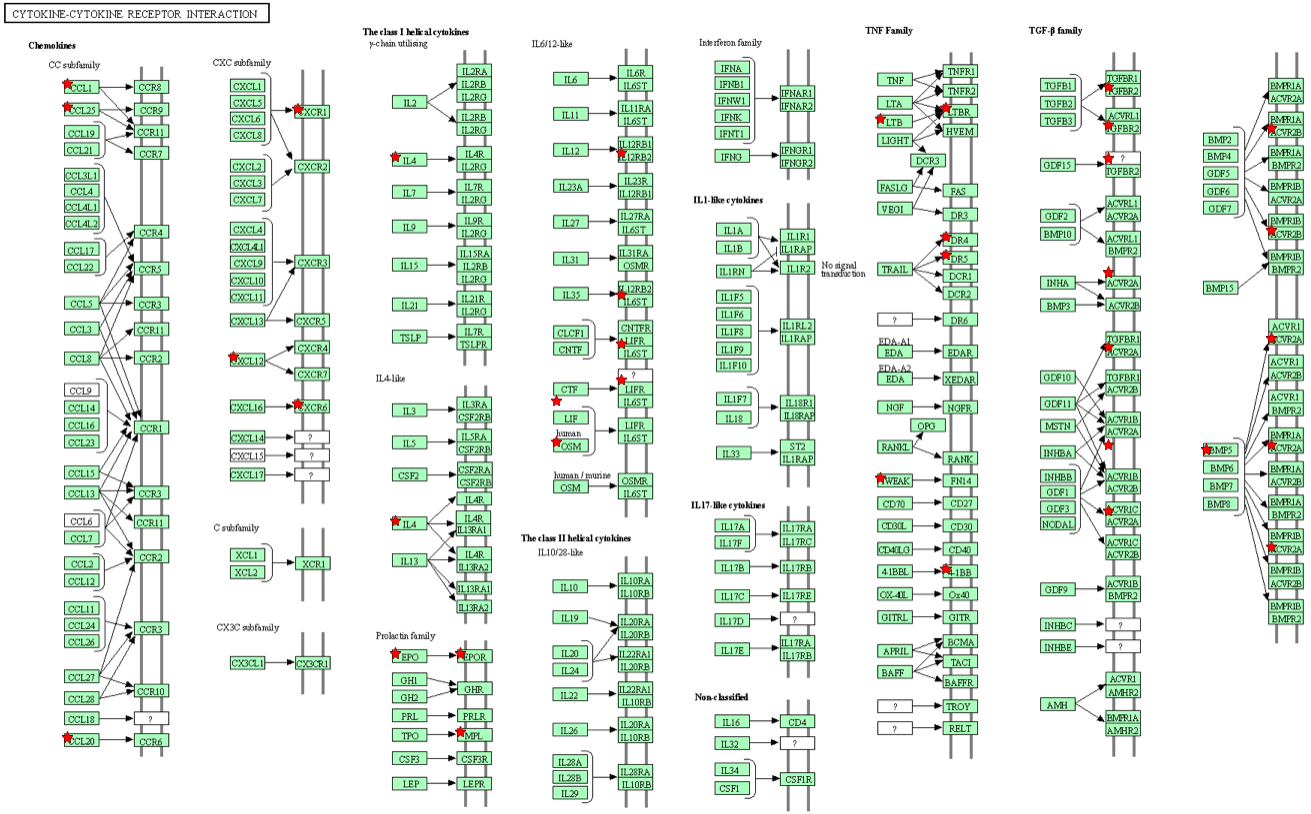

Supplement: Supplementary file 1 — Additional file 1: Figure S1. KEGG enrichment analysis of genes pertaining to the chemokine-mediated signaling pathways in RNA extracted from PBMCs collected from two positive patients for SARS-Cov-2 IgM. Genes coding for the star-marked elements, implied in various chemokine-mediated and chemokine-chemokine signaling pathways, were found overexpressed at the first blood draw and gradually decreased over time (p-value = 0.018). [file 12967_2022_3429_MOESM1_ESM.png]

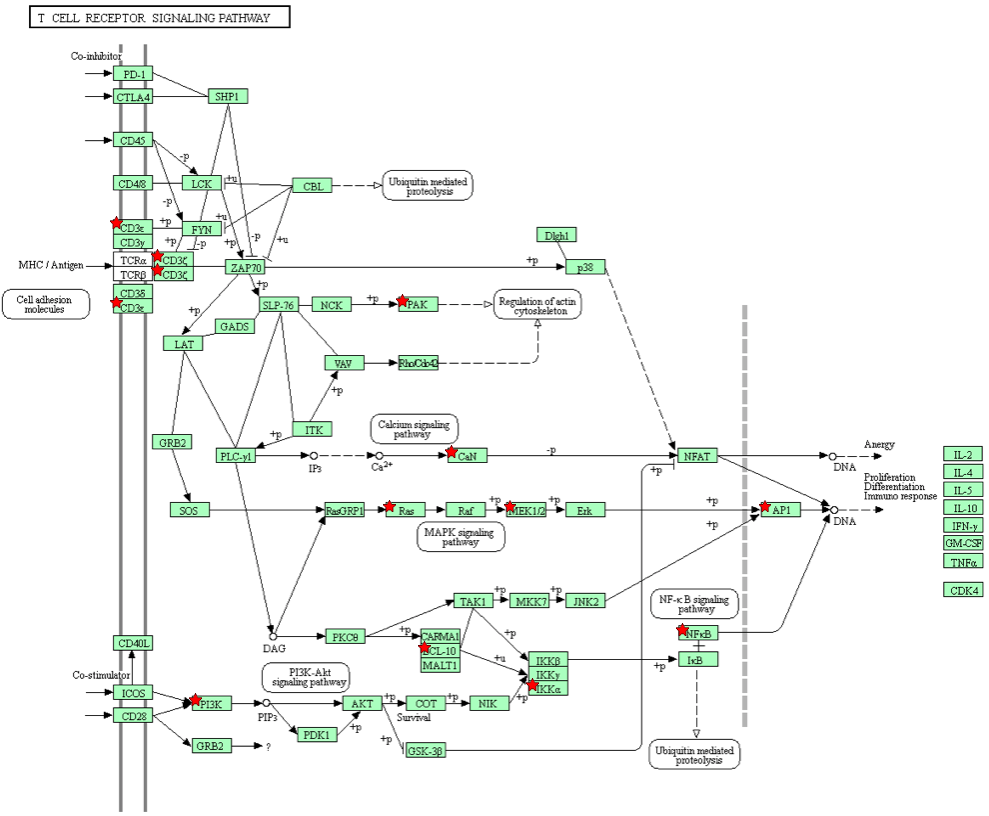

Supplement: Supplementary file 2 — Additional file 2: Figure S2. KEGG enrichment analysis of genes pertaining to the T-cell receptor signaling pathway in RNA extracted from PBMCs collected from two positive patients for SARS-Cov-2 IgM. Genes coding for the star-marked elements, implied in the T-cell receptor signaling pathway, were found to be increasingly upregulated in these patients over time (p-value = 0.017). [file 12967_2022_3429_MOESM2_ESM.png]

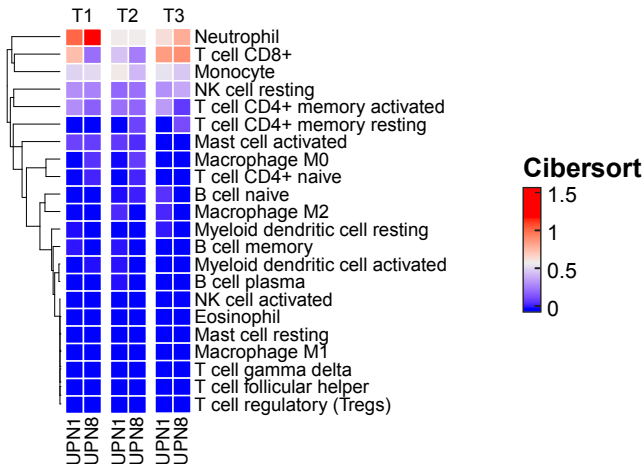

Supplement: Supplementary file 3 — Additional file 3: Figure S3. CYBERSORT analysis of PBMCs transcripts of samples collected from the two selected patients at baseline (T1), 2 months (T2), and 7 months (T3). [file 12967_2022_3429_MOESM3_ESM.pdf]
